# Systemic lupus erythematosus with diffuse splenic calcification: A case report

**DOI:** 10.1002/iid3.927

**Published:** 2023-07-25

**Authors:** Shanshan Su, Huohu Zhong

**Affiliations:** ^1^ Department of Ultrasound Second Affiliated Hospital of Fujian Medical University Quanzhou Fujian Province China

**Keywords:** case report, splenic calcification, systemic lupus erythematosus, ultrasonography

## Abstract

**Objective:**

Systemic lupus erythematosus (SLE) is a systemic autoimmune disease with the main clinical characteristics of multisystem and multiorgan involvement of the whole body. It is commonly seen in skin mucosa, skeletal muscle, and the respiratory system while rarely involving the spleen. In this case, we present a young female patient with SLE with the symptom of progressing splenic calcification.

**Methods:**

A 21–year–old female patient was admitted to our hospital in 2012 with complaints of “fever, abdominal pain, diarrhea, and knee pain”, and then was diagnosed with systemic lupus erythematosus combined with lupus nephritis. The first ultrasound examination was normal in 2012. However, when she returned to the hospital in 2016, she was found to have multiple calcifications in her spleen. After discharge, the patient was treated with commonly used immunosuppressive drugs and was followed up regularly for symptoms of multiple calcifications in the spleen.

**Results:**

Subsequent follow–up over a long period revealed a progressive development of multiple calcifications in the spleen, with a progressive increase in the size and number of calcified foci over time.

**Conclusion:**

When a patient is found to have diffuse splenic calcification on ultrasound, care should be taken to differentiate SLE with multiple splenic calcifications from sarcoidosis, tuberculosis, brucellosis, and rheumatoid arthritis in conjunction with a medical history and relevant laboratory tests.

## Graphical abstract

This case report aimed to report a medical curiosity that a young female patient with systemic lupus erythematosus with the symptom of progressing splenic calcification. To enhance the scientificity, we have been following her since 2012 and have kept a series of images. This paper summarizes the patient's disease progression, possible mechanisms, and related differential diagnosis.

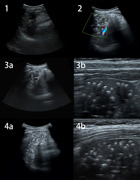

## 1 INTRODUCTION

Systemic lupus erythematosus (SLE) is an autoimmune inflammatory connective tissue disease involving multiple organs, most common in young females. The main clinical characteristics of SLE are the involvement of various systems and organs in the whole body, repeated recurrence and remission, and the presence of a large number of autoantibodies in the body.^1^, ^2^ It would cause irreversible damage to the affected organs and eventually lead to the patient's death if not treated in time. The aetiology of SLE still needs to be clarified. Currently, it is believed that under the interaction of genetic factors, environmental factors, estrogen level, and other factors, T lymphocytes decrease, T suppressor cell function declines, B cell hyperplasia, and a large number of autoantibodies are produced, which combine with the corresponding autoantigens in the body to form the related immune complex—deposited in the skin, joints, small blood vessels, glomerulus, and other places. With the participation of complement, it can cause acute and chronic inflammation and tissue necrosis, or antibodies directly interact with histocyte antigens and cause cell destruction, thus leading to multisystem damage to the body.^3^ The spleen is rarely involved in SLE and generally manifests as splenomegaly, spontaneous splenic rupture, low splenic function, and “onion‐skin lesions” characteristics of splenic arterioles. Spleen calcification is a feature of connective tissue disease, and the exact mechanism of diffuse spleen calcification is still unclear. Blocking Fc receptors by circulating immune complexes can lead to functional splenic dysfunction.^4^

We present a case with a history of SLE who subsequently developed diffuse spleen calcifications. In this given case, significant progress in spleen calcification was observed in ultrasound examinations from 2012 to 2023.

## 2 CASE REPORT

**Figure 1:**
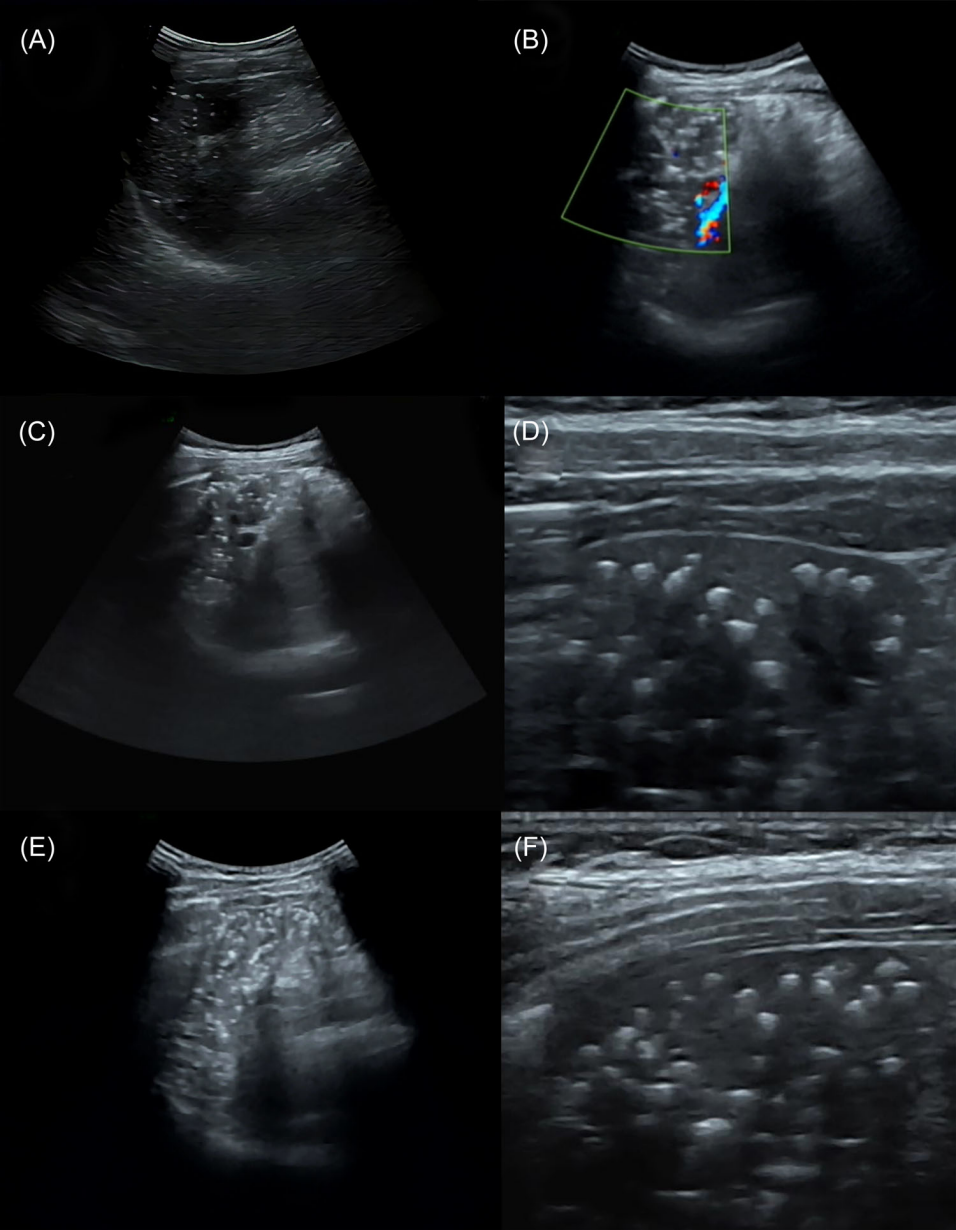
Ultrasound examination of the spleen from 2016 to 2023. (A) Ultrasound examination in 2016 indicated the parenchymal echo of spleen was uniform, and multiple punctiform strong echoes could be detected internally, with the maximum being about 3 mm, some of which were accompanied by the comet–tail sign; (B) Colorful ultrasound examination in 2020 showed that the number of punctiform strong echoes in the splenic parenchyma increased significantly compared with 2016, and the diameter increased slightly; (C, D): Ultrasound examination in 2021 demonstrated that the strong echoes in the splenic parenchyma have increased to the maximum diameter of 3.4 mm; (E, F): Ultrasound examination in 2023 indicated that the strong echoes in the splenic parenchyma increased significantly, with a maximum diameter of 3.7 mm.

A 21‐year‐old female patient was admitted to the hospital in 2012 with “fever, abdominal pain, diarrhoea, and knee soreness” as the chief complaint. The patient had a previous history of seafood allergy and no previous history of tuberculosis, rural areas, or contact with birds and poultry. Laboratory examination: ESR: 30 mm/H, complement C3:0.51 g/L (reference value 0.85–1.93 g/L), routine urine protein (+), ANA: spot type (positive). The colorful ultrasound examination of the digestive system showed no abnormality. In summary, the diagnosis was “systemic lupus erythematosus complicated by (1) lupus nephritis; (2) Intestinal vasculitis.” The patient was treated with immunomodulating therapy through methylprednisolone, hydroxychloroquine sulfate and mycophenolate mofetil, anti‐infection treatment through thiamphenicol and moxifloxacin, relieving spasms, preserving stomach, and replenishing fluid. They were then discharged with improvement. After discharge, common immunosuppressive drugs were taken, and the condition was well controlled. From 2016 to 2023, the patient was repeatedly hospitalized for “systemic lupus erythematosus acting.” Ultrasound examination of the spleen from 2016 to 2023 (Figure 1) indicated that the parenchymal echo was relatively uniform, and multiple punctiform strong echoes could be detected internally, with the maximum size of strong echoes increased over time from 3.0 to 3.7 mm. (the low‐frequency convex array probe obtained Figure 1C,E), the linear array probe got Figure 1D,F.

## 3 DISCUSSION

Characteristics of this case: ① A young woman with SLE with recurrent episodes of lupus nephritis and intestinal vasculitis. ② Diffuse calcification of the spleen is associated with the condition of SLE, developed from nothing, and the spleen calcification increases with repeated attacks of SLE.

SLE is a chronic autoimmune disease with less involvement of the spleen. Current reports mainly focus on splenomegaly, spontaneous splenic rupture, low splenic function, and “onion‐skin lesions” characteristics of splenic arterioles.^5^ Reports of long‐term follow‐up are even rarer. SLE patient with Diffuse splenic calcification associated is very sporadic, with no more than 30 cases reported worldwide. Tieng et al.^6^ summarized the clinical features and splenic calcification characteristics among 10 patients with SLE accompanied by diffuse splenic calcification and found that 9 out of 10 patients showed positive in ANA and dsDNA, and eight patients had lupus nephropathy; Splenic calcification in patients with SLE follows a unique pattern, demonstrated as discrete, rounded, small, widely distributed calcifications within the spleen but excluding the capsule and capsule area. Besides, calcifications' linear, tubular, or ovular morphology indicates possible vascular origin. Kitamura dissected a case of SLE with diffuse splenic calcification and found that the intrasplenic calcification nodules formed around the central splenic artery or arterioles, suggesting that this characteristic splenic calcification may be an extreme form of “onion‐skin lesions.”^7^ The splenic calcification formed after the treatment of granulomatous infection was extensive, often presented as spotty‐like, and relatively less quantity. Tuberculosis usually has a history of tuberculosis exposure, often accompanied by pulmonary manifestations, tuberculin test, and TB T cell test positive; Brucellosis often has a history of pastoral contact; Calcification in other connective tissue diseases is more isolated, less dense, and closer to the capsule area.^6^

This case's clinical and sonographic features are consistent with those described in the literature. It is worth noting that the splenic calcification of the patient, in this case, was accompanied by multiple SLE activities from 2012 to 2023, which showed a process from inexistence to existence, from less to more, indicating that numerous splenic calcifications may be a secondary result of immune‐mediated arterial inflammation related to repeated SLE episodes.^8^ The link between high levels of anti‐DNA antibodies and disease activity has been further established.^9^

## 4 CONCLUSION

Ultrasound diagnosis for SLE with diffuse splenic calcifications should be differentiated from granulomatous disease, tuberculosis, brucellosis, rheumatoid arthritis, and so forth. But these diseases can not be well identified by ultrasound and other imaging examinations alone, medical history and relevant laboratory examinations should be combined.

## AUTHOR CONTRIBUTIONS

**Shanshan Su**: Conceptualization (lead); validation; investigation; resources; writing—review & editing (equal), supervision, project administration and funding acquisition; **Huohu Zhong**: Conceptualization (supporting)—methodology—formal analysis—data curation—writing—original draft, writing—review & editing (equal) and visualization.

## CONFLICT OF INTEREST STATEMENT

The authors declare no conflict of interest.

## Data Availability

The data that support the findings of this study are available from the corresponding author upon reasonable request.
